# Mutations in *SETD2* and genes affecting histone H3K36 methylation target hemispheric high-grade gliomas

**DOI:** 10.1007/s00401-013-1095-8

**Published:** 2013-02-16

**Authors:** Adam M. Fontebasso, Jeremy Schwartzentruber, Dong-Anh Khuong-Quang, Xiao-Yang Liu, Dominik Sturm, Andrey Korshunov, David T. W. Jones, Hendrik Witt, Marcel Kool, Steffen Albrecht, Adam Fleming, Djihad Hadjadj, Stephan Busche, Pierre Lepage, Alexandre Montpetit, Alfredo Staffa, Noha Gerges, Magdalena Zakrzewska, Krzystof Zakrzewski, Pawel P. Liberski, Peter Hauser, Miklos Garami, Almos Klekner, Laszlo Bognar, Gelareh Zadeh, Damien Faury, Stefan M. Pfister, Nada Jabado, Jacek Majewski

**Affiliations:** 1 Division of Experimental Medicine, McGill University and McGill University Health Centre, Montreal, QC Canada; 2 McGill University and Genome Quebec Innovation Centre, Montreal, QC Canada; 3 Department of Human Genetics, McGill University and McGill University Health Centre, Montreal, QC Canada; 4 Division of Pediatric Neurooncology, German Cancer Research Center (DKFZ), Heidelberg, Germany; 5 Clinical Cooperation Unit Neuropathology, German Cancer Research Center (DKFZ), Heidelberg, Germany; 6 Department of Paediatric Oncology, Hematology and Immunology, Heidelberg University Hospital, Heidelberg, Germany; 7 Department of Pathology, Montreal Children’s Hospital, McGill University Health Centre, Montreal, QC Canada; 8 Division of Hemato-Oncology, Montreal Children’s Hospital, McGill University Health Centre, Montreal, QC Canada; 9 Department of Molecular Pathology and Neuropathology, Medical University of Lodz, Lodz, Poland; 10 Department of Neurosurgery, Polish Mother’s Memorial Hospital Research Institute, Lodz, Poland; 11 2nd Department of Paediatrics, Semmelweis University, Budapest, Hungary; 12 Department of Neurosurgery, Medical and Health Science Center, University of Debrecen, Debrecen, Hungary; 13 Division of Neurosurgery, Toronto Western Hospital, Ontario, Canada; 14 Department of Paediatrics, The Research Institute of the McGill University Health Centre, McGill University, Montreal, QC Canada

**Keywords:** High-grade glioma, H3K36 methylation, SETD2, Epigenetic, Pediatric, Young adult

## Abstract

Recurrent mutations affecting the histone H3.3 residues Lys27 or indirectly Lys36 are frequent drivers of pediatric high-grade gliomas (over 30 % of HGGs). To identify additional driver mutations in HGGs, we investigated a cohort of 60 pediatric HGGs using whole-exome sequencing (WES) and compared them to 543 exomes from non-cancer control samples. We identified mutations in *SETD2*, a H3K36 trimethyltransferase, in 15 % of pediatric HGGs, a result that was genome-wide significant (FDR = 0.029). Most *SETD2* alterations were truncating mutations. Sequencing the gene in this cohort and another validation cohort (123 gliomas from all ages and grades) showed *SETD2* mutations to be specific to high-grade tumors affecting 15 % of pediatric HGGs (11/73) and 8 % of adult HGGs (5/65) while no *SETD2* mutations were identified in low-grade diffuse gliomas (0/45). Furthermore, *SETD2* mutations were mutually exclusive with *H3F3A* mutations in HGGs (*P* = 0.0492) while they partly overlapped with *IDH1* mutations (4/14), and *SETD2*-mutant tumors were found exclusively in the cerebral hemispheres (*P* = 0.0055). SETD2 is the only H3K36 trimethyltransferase in humans, and *SETD2*-mutant tumors showed a substantial decrease in H3K36me3 levels (*P* < 0.001), indicating that the mutations are loss-of-function. These data suggest that loss-of-function SETD2 mutations occur in older children and young adults and are specific to HGG of the cerebral cortex, similar to the H3.3 G34R/V and IDH mutations. Taken together, our results suggest that mutations disrupting the histone code at H3K36, including H3.3 G34R/V, IDH1 and/or SETD2 mutations, are central to the genesis of hemispheric HGGs in older children and young adults.

**Electronic supplementary material:**

The online version of this article (doi:10.1007/s00401-013-1095-8) contains supplementary material, which is available to authorized users.

## Introduction

Malignant primary brain and central nervous system (CNS) tumors occur at an age-adjusted incidence rate of 7.3 out of 100,000 people across all ages and are the leading cause of cancer-related death in children [9]. High-grade gliomas [HGG; grade III and grade IV astrocytomas/glioblastoma (GBM)] are highly aggressive and deadly brain tumors [9, 32] and are more commonly diagnosed in adults. GBM remains essentially incurable despite decades of concerted therapeutic efforts [5]. One impediment to treatment is that GBM is diagnosed as a single pathological entity, which cannot discriminate potential genetic drivers and molecular subtypes. This impacts the design and outcome of clinical trials and likely contributes to the apparent inherent resistance of GBM to adjuvant therapies. Because of the similar histology, current treatments for GBM in children are driven by adult studies and show, as in adults, little therapeutic success.

We and others recently identified two recurrent mutations in *H3F3A,* which encodes the replication-independent histone 3 variant H3.3, in over 30 % of pediatric and young adult GBM [32, 42]. The mutations, K27M and G34R/G34V, occur at positions in the histone tail that are critical for post-translational modifications involved in the histone code, which determines chromatin structure and gene expression. H3.3 K27M mutations were also identified in over 70 % of pediatric diffuse intrinsic pontine glioma (DIPG), a fatal HGG of the brainstem [18, 42] as well as K27M mutations in the canonical H3.1 in 18 % of samples [42]. H3.3 mutations significantly overlapped with mutations in *TP53* and in *ATRX* (α-thalassemia/mental-retardation syndrome-X-linked) [13, 40] and less frequently with the ATRX hetero-dimer *DAXX*, which encode subunits of a chromatin remodeling complex required for H3.3 incorporation at pericentric heterochromatin and telomeres [8, 13]. H3.3 mutations represent the pediatric counterpart of the recurrent hotspot mutations in isocitrate dehydrogenase 1 or 2 (*IDH1/2*) [27, 44]. *IDH1* R132 mutations are gain-of-function, causing the enzyme to produce 2-hydroxyglutarate (2-HG) [7, 27] and *IDH1*-mutant tumors display distinct DNA methylation profiles with global hypermethylation, termed a glioma-CpG island methylator phenotype (G-CIMP) [26, 35, 38]. Interestingly, similar to pediatric GBM, *IDH1* mutations were shown to occur in association with *TP53* [3, 27] and *ATRX* mutations in adult diffuse astrocytic tumors [14, 16, 25], illustrating an important constellation of mutations in the development of pediatric and secondary GBM. In the present study, we sought to identify drivers of HGG in pediatric samples that did not carry H3.3 or IDH mutations. We investigated a cohort of 60 pediatric HGGs utilizing statistical analysis of whole-exome sequencing (WES) on a genome-wide ranking scale and validated results in an independent validation cohort of 123 gliomas from all ages and grades. Herein, we present data showing the importance and functional impact of mutations in the H3K36 trimethyltransferase *SETD2* in HGGs of the cerebral hemispheres.

## Materials and methods

### Sample characteristics and pathological review

Samples were obtained with informed consent following approval of the Institutional Review Board of individual hospitals. Samples were reviewed by senior neuropathologists (S.A., A.K.) according to WHO guidelines. Fifty-one pediatric grade IV astrocytomas (glioblastoma, GBM) patients and nine pediatric grade III astrocytomas from patients aged 1–20 years were analyzed by whole-exome sequencing (44 previously published in [32]). An additional 123 adult and pediatric gliomas of diverse histology and grade were also included for targeted sequencing of *SETD2, IDH1* and *H3F3A*. Available clinical and relevant mutational characteristics are detailed in Table S1. Tissues were obtained from the London/Ontario Tumor Bank, the Pediatric Cooperative Health Tissue Network, the Children’s Oncology Group, The Montreal Children’s Hospital and from collaborators in Poland, Hungary and Germany.

### DNA extraction

Genomic DNA was extracted from frozen tumor tissue utilizing the Qiagen DNeasy Blood and Tissue kit according to instructions from the manufacturer (Qiagen).

### Alignment and variant calling for whole-exome sequencing

Standard instructions from the manufacturer were used for target capture with the Illumina TruSeq exome enrichment kit and 100 bp paired-end sequencing reads on the Illumina HiSeq platform with bioinformatic processing and variant annotation as previously described [32]. For the selected genes of interest shown in Table S1, variants in these genes that were private to tumor samples are shown, i.e. those variants not seen within the 1000 genomes (http://www.1000genomes.org/) or NHLBI exome (http://evs.gs.washington.edu/EVS/) databases, or in any of our 543 control exomes. Missense mutations were highlighted if they occurred within highly conserved residues in vertebrates, assessed utilizing the UCSC Genome Browser (http://genome.ucsc.edu/) conservation track tool [17]. To assess significance of mutations in our tumor dataset, we used a case–control approach to compare the frequency of private mutations in each gene in the 60 tumor exomes to 543 control exomes, which were from constitutional DNA of patients with Mendelian diseases also sequenced at the McGill University and Genome Quebec Innovation Centre (Table S2). We controlled for false discovery rate using the Benjamini–Hochberg procedure. All variants in these genes are detailed in Table S2, whereas only private variants, likely to be somatic, and in highly conserved residues (likely to impact function), are highlighted in Table S1 and discussed in this study.

### Targeted next-generation sequencing of *SETD2*

Coding regions of *SETD2* were amplified using the Fluidigm. Access Array system (http://www.fluidigm.com/access-array-system.html) and sequenced on a half run of the GS FLX Titanium system from Roche 454. Forty pairs of primers were designed to cover all coding regions of the 21 exons of *SETD2*. Primers were designed using Primer3 (http://frodo.wi.mit.edu/primer3/) [28]. The parameters were set to achieve melting temperatures ranging from 57 to 59 °C. Lengths of PCR products are between 197 and 394 bp. The UCSC Genome Browser (http://genome.ucsc.edu/) was used to download target genomic regions prior to design and identify variants (based on dbSNP135: http://www.ncbi.nlm.nih.gov/projects/SNP/) [17]. PCRs were performed on 48 × 48 IFC (Integrated Fluidic Circuit) chips. On each chip, 40 regions were amplified in 48 samples. Amplification of target regions and addition of 454 sequencing adapters and individual bar codes occur in the same PCR performed on the Fluidigm FC1 cycler. All samples were individually bar coded and sequenced in one half-region of a GS FLX Titanium run. Validation of variants was done with Sanger sequencing. Following this, statistical analyses of Fisher’s exact test for contingency comparisons were performed utilizing GraphPad Prism 5 software.

### Immunoblotting analysis of H3K36me3 levels in patient tumors

Fresh-frozen tumor tissues with adequate material and known *SETD2*, *H3F3A* and *IDH1* mutational status were lysed using the EpiQuik Total Histone Extraction Kit (Epigentek, USA). Lysates were quantified utilizing standard BioRad protein assay (BioRad) and loaded onto 15 % acrylamide gels and run for 2 h at 100 V. Proteins were transferred to polyvinylidene difluoride (PVDF) membranes at room temperature for 5 min, using the Trans-Blot Turbo transfer system (BioRad) at LOW MW setting, blocked and immunoblotted with the following conditions overnight at 4 °C: rabbit polyclonal anti-H3K36me3 (Abcam #9050) at 1:1,000 in 5 % skim milk and rabbit polyclonal anti-H3 (Abcam #1791) at 1:1,000 in 5 % skim milk. Membranes were subsequently washed thrice with tris-buffered saline-Tween 20 (TBS-T) and incubated with horseradish peroxidase (HRP)-conjugated donkey anti-rabbit IgG secondary antibody (GE Healthcare #NA934V) at 1:5,000 with Precision Protein StrepTactin-HRP conjugate at 1:10,000 (BioRad #161-0380) in 5 % skim milk for 1 h at room temperature and revealed utilizing Amersham ECL detection (Amersham Biosciences). H3K36me3 bands from four independent blots were quantified utilizing ImageQuant TL v2003.02 (Amersham Biosciences), normalized to total H3, and normalized ratios were compared statistically using two-tailed *T* test for significance.

### Methylation array profiling

DNA extracted from a subset of pediatric HGGs demonstrating defects in *SETD2*, *IDH1,* or *H3F3A* at G34 and wild-type tumors (*n* = 36) was analyzed for genome-wide DNA methylation patterns utilizing the HumanMethylation450 BeadChip according to the manufacturer’s instructions (Illumina, San Diego, USA) at the McGill University and Genome Quebec Innovation Centre. Of the >480,000 probes on the methylation chip, we discarded probes with ≥90 % sequence similarity to multiple genomic locations, with sequence variants in the probe binding region (1000 Genomes Project, SNPs with a minor allele frequency ≥2/120), and probes located on sex chromosomes, leaving 392,904 autosomal probes for further analysis. Subset-quantile within array normalization was performed on beta values using the SWAN method [23]. For unsupervised hierarchical clustering, the top 8,000 most variable probes (by standard deviation) were utilized with average linkage and Pearson correlation algorithms across the dataset. Consensus clustering was performed utilizing the *k*-means algorithm with 1,000 iterations on the top 8,000 most variable probes in the dataset. Methylation analysis was performed utilizing R (R version 2.14.2, http://cran.r-project.org/) with Minfi and ConsensusClusterPlus loaded packages.

## Results and discussion

### *SETD2* mutations affect a significant proportion of pediatric HGGs

**Fig. 1:**
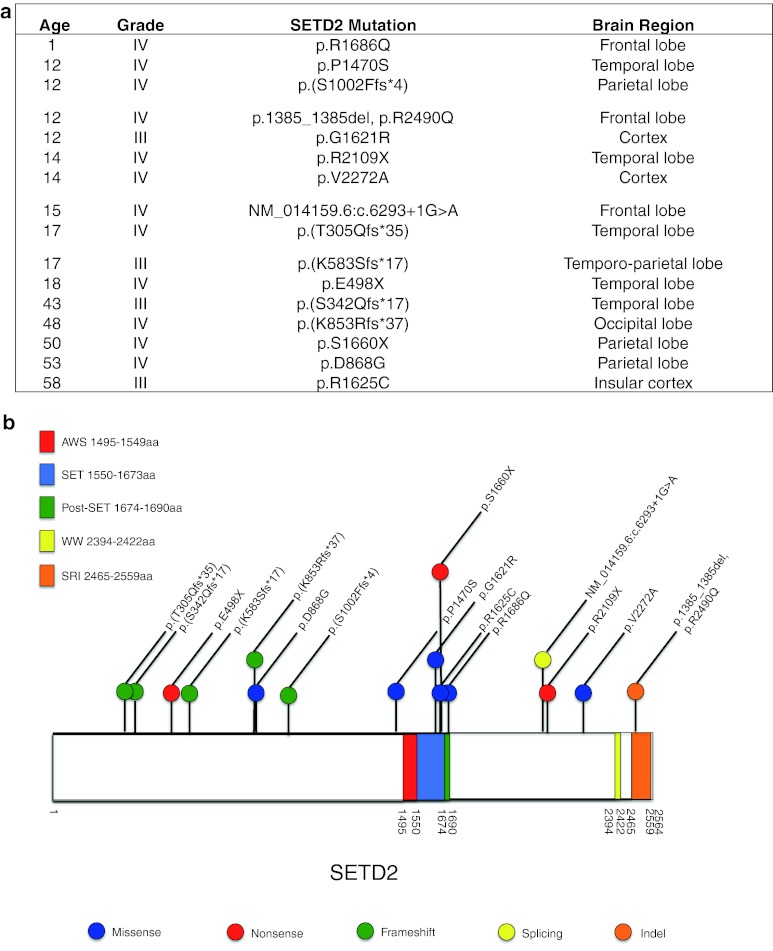
Missense/truncating mutations of the H3K36 trimethyltransferase *SETD2* identified in pediatric and adult high-grade gliomas. **a** Patient age, tumor grade, and affected brain region of tumors with *SETD2* mutation. **b** Schematic mapping type and distribution of missense/truncating mutations in SETD2 in 183 gliomas included in the study

To identify genetic drivers in samples not carrying mutations in *IDH1* and *H3F3A*, we analyzed 60 pediatric HGG tumors [grades III (*n* = 9) and IV (*n* = 51)] using WES (44 previously reported; Table S1) [32]. As matched normal DNA was unavailable for the majority of tumors, we identified private mutations that were present in tumors but were absent from public databases (1000 genomes project [24], NHLBI exomes) and from our set of 543 control exomes, and considered these as candidate somatic mutations. We compared the frequency of private mutations in each gene between the 60 tumors and 543 controls using Fisher’s exact test and used a false discovery rate threshold (FDR) of 0.05 to correct for multiple tests. Our case–control approach effectively corrects for the background rate of mutations in each gene (which implicitly includes the length of the gene and the mutability). We filtered out variants that were predicted to be tolerated/benign/unknown by both SIFT and PolyPhen-2 [1], and identified private mutations that we considered as candidate somatic mutations. The top genes by mutation frequency are shown in Table S2. As expected, four genes previously associated with pediatric HGG showed a highly significantly number of mutations (*TP53*, *H3F3A*, *ATRX*, *NF1*) [32] (Table S2). In addition, two genes not previously reported in HGG, *SETD2* and *CSMD3*, achieved genome-wide significance (FDR = 0.029 and 0.031, respectively). For *SETD2*, this significance was more striking when only truncating mutations were considered (FDR = 0.0017), as no truncating mutations were seen in 543 controls, but tumor samples had frameshift (3), nonsense (1), and splicing (1) variants. In addition, the three missense variants in tumor samples occurred at highly conserved residues and were computationally predicted as damaging by both SIFT and Polyphen scores (Fig. 1a, b). In contrast, of the seven private variants in the control samples (all missense), only one is predicted damaging by both SIFT and Polyphen. H3.3 mutations occur at two positions within the histone tail involved in key regulatory post-translational modifications, K27 (directly) and K36 (indirectly). Driver loss-of-function *SETD2* mutations have recently been identified in two high-grade cancers, renal cell carcinoma [6, 12] and early T-cell precursor acute lymphoblastic leukemia [37]. The other candidate gene, *CSMD3* is expressed in adult and fetal brains; however, its functions are yet unclear [33]. Hence, we focused our next efforts on *SETD2* as the top candidate gene.

### *SETD2* mutations affect pediatric and adult HGGs of the cerebral hemispheres

We expanded our sequencing analysis and next sequenced *SETD2* in 123 additional gliomas of various ages and grades (Table 1; Table S1). Combining the discovery and the validation datasets, *SETD2* mutations were identified in a total of 15 % of pediatric HGG (11/73) and 8 % of adult HGG (5/65), and were not seen in low-grade diffuse gliomas (0/45) (*P* = 0.0133; Table 1; Table S1). Except for one sample, all mutations occurred in children above the age of 12, in adolescents and in younger adults, mirroring the age range of H3.3 G34R/V and IDH1 mutations in HGG (Fig. 1a; Figure S1) [18–21, 32, 35]. Notably, all tumors carrying *SETD2* mutations were localized in the cerebral hemispheres (*P* = 0.0055). *SETD2* mutations were mutually exclusive with *H3F3A* mutations (*P* = 0.049) in HGGs (0/70), but showed partial overlap with *IDH1* R132 mutations (4/14), *TP53* (4/8) and *ATRX* (3/9) mutations (Table S1).

**Table 1:** Frequencies of SETD2 mutations in 183 pediatric and adult gliomas

Glioma	Mutated	Wild type	Total	Frequency (%)
Grade IV	12	85	97	12.37
Pediatric	9	51	60	15
Adult	3	34	37	8.11
Grade III	4	37	41	9.76
Pediatric	2	11	13	15.38
Adult	2	26	28	7.14
Grade II	0	45	45	0
Pediatric	0	23	23	0
Adult	0	22	22	0
Overall gliomas	16	167	183	8.7

### Missense/truncating mutations in *SETD2* impair trimethyltransferase activity of the enzyme and confer distinct global DNA methylation signatures

**Fig. 2:**
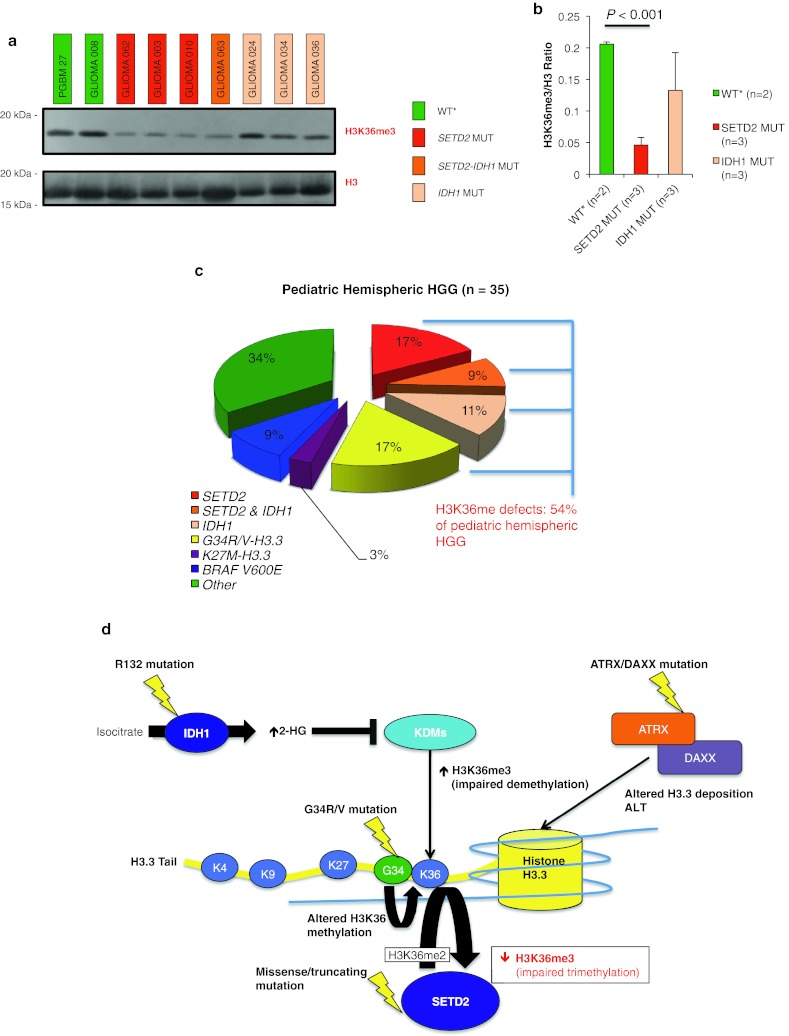
Missense/truncating mutations in *SETD2* impair H3K36 trimethyltransferase activity of the enzyme. **a** Western blot analysis of histone acidic extracts of *SETD2*-mutant tumor samples demonstrating a significant decrease in H3K36me3 levels, indicating impaired H3K36 trimethyltransferase activity of the enzyme. **b** Densitometric quantification of H3K36me3 levels assessed in four independent blots demonstrating a significant decrease in H3K36me3/Total H3 normalized ratios in *SETD2*-mutant tumors. WT* = WT for *SETD2*, *IDH1* and *H3F3A*. **c** Pie representation of mutations directly or indirectly affecting H3K36 methylation (H3K36me) in pediatric HGGs of the cerebral hemispheres (*n* = 35) indicating that approximately half of these tumors display defects, pointing to H3K36 dysregulation as a critical mechanism of hemispheric high-grade gliomagenesis. **d** Schematic representation of major genetic and epigenetic defects leading to altered H3K36 methylation in hemispheric HGGs

*SETD2* encodes the only H3K36 trimethyltransferase in humans [11, 41]. To support computational predictions of the damaging nature of *SETD2* mutations, we assessed H3K36 trimethyltransferase activity in histone acidic extractions of patient tissue samples through Western blotting for H3K36me3 levels, an indicator of *SETD2* activity [11]. Immunoblot analysis revealed a significant decrease in total H3K36me3 levels in *SETD2*-mutant gliomas (Fig. 2a), as well as a significantly decreased normalized ratio of H3K36me3 to total H3 levels in *SETD2*-mutant tumors (*P* < 0.001; Fig. 2b) showing loss-of-function as a result of *SETD2* missense/truncating mutations.

**Fig. 3:**
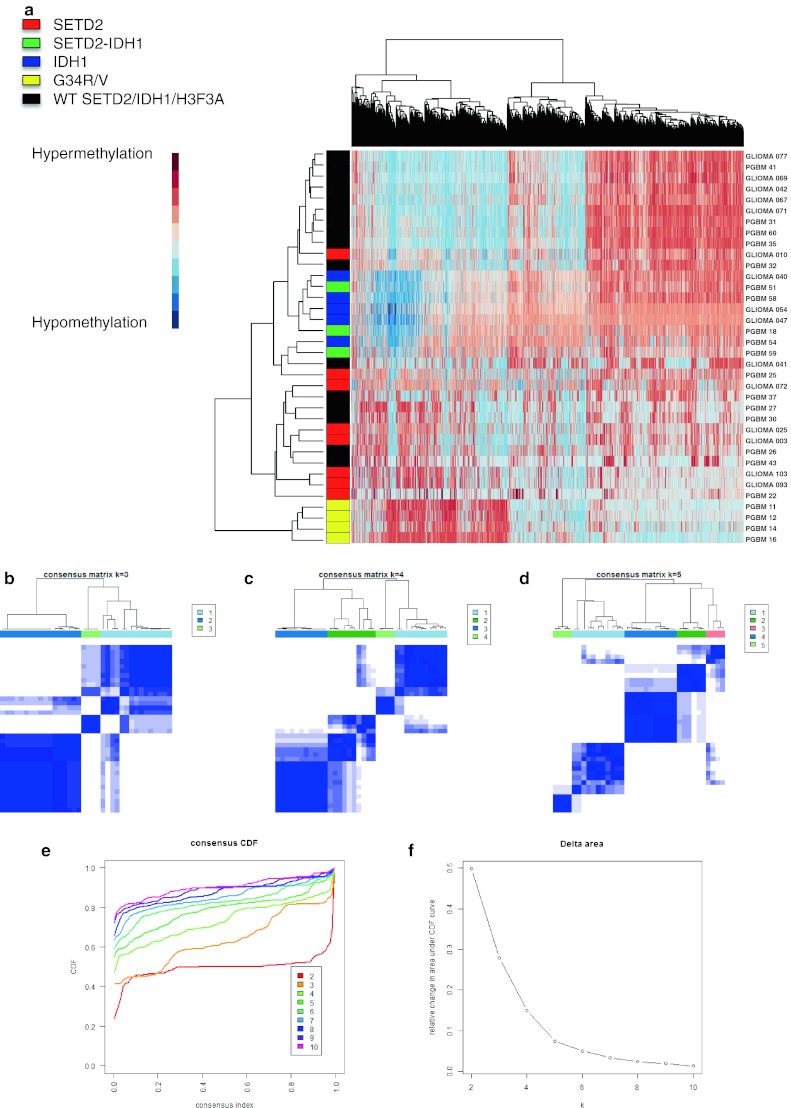
Mutations affecting H3K36 methylation confer distinct global DNA methylation signatures. **a** Unsupervised hierarchical clustering of methylation Beta-values representing the top 8,000 most variable probes between samples mutated for *SETD2*, *IDH1* or H3.3 G34R/V and high-grade gliomas wild-type (WT) for these genes (*n* = 36). **b** *k*-means consensus matrices for *k* = 3 (**b**), *k* = 4 (**c**) or *k* = 5 (**d**) for the top 8,000 most variable probes. **e** Empirical cumulative distribution function (CDF) plot and delta area differences (**f**) for indicated numbers of clusters (*k* = 2 to *k* = 10)

GBMs with epigenetic driver mutations such as H3.3 K27M or G34R/V, as well as those with IDH1 mutations, display distinct DNA methylation profiles and clinical characteristics. They also arise in distinct anatomic compartments, with *IDH1*- and H3.3 G34R/V-mutant tumors being restricted to areas of the cerebral hemispheres [18–21, 32, 35]. We and others have previously described the distinct heterogeneity of epigenetic profiles underlying HGGs including GBM [2, 35, 36]. We thus sought to characterize the DNA methylation profiles of 36 pediatric HGG tumors with mutations likely to affect K36 methylation status, using the Illumina 450K array platform as previously described [35]. *SETD2* mutations yielded global DNA methylation patterns distinct from tumors with H3.3 G34R/V mutations, but which partly overlapped with *IDH1*-mutant methylation patterns (Fig. 3a–f). Notably, promoters at *OLIG1/2* loci, characteristically hypermethylated in G34R/V-mutated samples, were not hypermethylated in *SETD2* mutants (Figure S2) [2, 35].

### Mutations identified in candidate oncogenic drivers and other genes involved in histone post-translational modifications in HGGs

We further investigated our dataset for mutations in other genes affecting PTM of H3K27 or H3K36 but which did not reach the statistically significant mutation levels. Eight distinct mammalian enzymes methylate H3K36 and share the catalytic SET domain, but have varying preferences for K36 residues in different methylation states (reviewed in [41]). SETD2 is the only enzyme in humans to catalyze H3K36 tri-methylation [11], while its mono- and/or di-methylation is catalyzed by NSD1, NSD2, NSD3, SETMAR, ASH1L, SMYD2 or SETD3 (reviewed in [41]). We identified one missense and one nonsense mutation in *ASH1L* (concurrently with *SETD2* mutation) and *SETD3* (1 missense mutation concurrently with *SETD2* mutation). One PGBM mutant for *SETD2* also had a missense mutation in *UTX/KDM6A* (H3K27 demethylase). This same PGBM had a missense mutation in *PBRM1,* a gene frequently mutated in renal cell carcinoma in association with *SETD2* [39]. We also identified two mutations in *KDM5C* (H3K4 demethylase) (Table S1). Interestingly, no mutations in the cancer-implicated histone methyltransferase *EZH2* were identified. Mutations in these genes were not prevalent enough to be statistically associated with HGGs in our sample set; however, it remains possible that they contribute to pathogenesis in a small fraction of HGG cases.

Further investigation of the exome dataset revealed previously described mutations in *BRAF* (V600E [30, 31], 5/60 pediatric HGG), which did not overlap with the epigenetic driver mutations we identify (Table S1; Fig. 2c). Other alterations also previously described in GBM, which may provide pathways alternative or complementary to epigenomic dysregulation, included *PTEN* mutations (two samples) which overlapped with H3.3 K27M while *EGFR* mutation or amplification (three samples) and *CDKN2A* mutation/loss (five samples) partially overlapped with *SETD2* mutations (Table S1). Truncating mutations in the mismatch repair genes [10] *MSH6* (three samples) and *MSH2* (one sample) were identified and were concurrent with *IDH1* (two samples) and *SETD2* (three samples) mutations. Of note, *SETD2* mutations were absent in a large cohort of 125 cases of medulloblastoma [15] another major group of pediatric brain tumors.

### Alteration of H3K36 post-translational modifications characterize hemispheric adolescent and younger adult HGG

Post-translational modification of resident histones modulates the properties of chromatin, impacting cell state and differentiation and determining the outcome of virtually all DNA processes in eukaryotes. Methylation of H3K36 is a key histone mark and has been widely associated with active chromatin but also with transcriptional repression, alternative splicing, DNA replication and repair, DNA methylation and the transmission of memory of gene expression from parents to offspring during development (reviewed in [41]). We identify loss-of-function mutations in *SETD2,* in 15 % of pediatric and 8 % of adult high-grade gliomas in a cohort of 183 samples from all ages and grades II–IV of glioma (Fig. 1a; Table S1). We further show *SETD2* mutations to be specific to high-grade tumors (*P* = 0.013), to HGGs located within the cerebral hemispheres (*P* = 0.005), and to be mutually exclusive with H3.3 mutations we [18, 32] and others [42] previously identified in pediatric high-grade astrocytomas (*P* = 0.049). *SETD2* alterations overlapped with *IDH1* mutations in 4 of 14 tumors (Table S1). Strikingly, the oncometabolite produced by *IDH1* mutations inhibits a plethora of histone demethylases (KDMs) causing aberrant histone methylation at defined residues including K27 and K36 and a block to cell differentiation [4, 22, 29, 43]. We [21] and others [14] have previously shown the association of *ATRX* and *TP53* mutations in *IDH1*-mutant diffuse astrocytic gliomas, and others have pointed to mutations in *CIC* and 1p19q loss in *IDH1*-mutant oligodendroglial tumors. Thus, *IDH1* mutations may require other key genetic events in a specific context for full-blown tumorigenesis, which may include *SETD2* mutations as suggested by our cohort. H3.3K36 methylation can be thus disrupted by H3.3 G34R/V mutation, IDH mutations and the *SETD2* mutations we report herein (Fig. 2a, b). Furthermore, our current analysis suggests that this histone mark is specifically altered in hemispheric adolescent and younger adult HGG (Fig. 2c, d) [2, 18, 19, 25, 32, 34, 35], and that the functional effect differs between *SETD2* and H3.3 mutations (Fig. 3; Figure S2). Future studies directed towards elucidating the importance of H3K36 methylation in cortical astrocytes and neural progenitor cells, and its dysregulation in tumorigenesis may lend insight into the regional specificity of these defects, while improved understanding of the consequences of altered chromatin remodeling induced by these mutations will help guide alternative therapeutic avenues for these deadly cancers.

## Electronic supplementary material

Below is the link to the electronic supplementary material.

Supplementary material 1 (PDF 119 kb)

Supplementary material 2 (PDF 51 kb)

Supplementary material 3 (PDF 116 kb)
