# Supplementary material for: Mutations in SETD2 and genes affecting histone H3K36 methylation target hemispheric high-grade gliomas
Source: Acta Neuropathol. 2013 Feb 16;125(5):659–69. doi: 10.1007/s00401-013-1095-8 (PMC3631313; doi:10.1007/s00401-013-1095-8)
Supplement: Supplementary file 1 — Supplementary material 1 (PDF 119 kb) [file 401_2013_1095_MOESM1_ESM.pdf]

Table S1: Clinical and molecular characteristics of 183 pediatric and adult gliomas included in the study.

Sample ID	WES	Pediatric/Adult	Age (years)	Tumor location	Sex	Type	Grade	H3.3	*ATRX/DAXX*	*TP53*	*IDH1* $	*SETD2* #	Other Histone 3 Modifying Genes *, §	EGFR Mutation/Amplification ¥	BRAF V600E	*SMARCB1*	*CDKN2A* Mutation/Deletion £	*MSH2, MSH6*	*PTEN*	*PIK3CA*
**PGBM 01**	Yes	Pediatric	13	Brainstem	F	Glioblastoma	IV	K27M	p.C1122fs	P152fs; R306X	WT	WT	WT	WT	WT	WT	WT	WT	WT	WT
**PGBM 02**	Yes	Pediatric	8	Temporo-parietal lobe	M	Glioblastoma	IV	K27M	WT	R213X	WT	WT	WT	WT	WT	WT	WT	WT	WT	WT
**PGBM 03**	Yes	Pediatric	12.5	Ventricle	M	Glioblastoma	IV	K27M	WT	N131del	WT	WT	WT	WT	WT	WT	WT	WT	p.G132A	WT
**PGBM 04**	Yes	Pediatric	10	Thalamus and lateral ventricle	M	Glioblastoma	IV	K27M	p.K1057fs %	G262fs	WT	WT	WT	WT	WT	WT	WT	WT	WT	WT
**PGBM 05**	Yes	Pediatric	9	NA	F	Glioblastoma	IV	K27M	WT	WT	WT	WT	KDM5C: p.Y113X	WT	WT	WT	WT	WT	WT	WT
**PGBM 06**	Yes	Pediatric	11	Thalamus	M	Glioblastoma	IV	K27M	p.M1800T %	WT	WT	WT	WT	WT	WT	WT	WT	WT	c.635-2A>C (NM_000314)	WT
**PGBM 08**	Yes	Pediatric	6	NA	F	Glioblastoma	IV	K27M	WT	R273C; R141C	WT	WT	WT	WT	WT	WT	WT	WT	WT	WT
**PGBM 09**	Yes	Pediatric	8	NA	F	Glioblastoma	IV	K27M	WT	R273P	WT	WT	KDM4C: p.D339G	WT	WT	WT	WT	WT	WT	WT
**PGBM 10**	Yes	Pediatric	11	NA	M	Glioblastoma	IV	K27M	WT	WT	WT	WT	WT	WT	WT	WT	WT	WT	WT	WT
**PGBM 11**	Yes	Pediatric	13	Frontal lobe	M	Glioblastoma	IV	G34R	p.S1394fs %	Y163C	WT	WT	WT	WT	WT	WT	WT	WT	WT	WT
**PGBM 12**	Yes	Pediatric	14	Temporal lobe	M	Glioblastoma	IV	G34R	p.E1727fs	R342X; R175H	WT	WT	WT	WT	WT	WT	WT	WT	WT	WT
**PGBM 13**	Yes	Pediatric	14	Occipital lobe	M	Glioblastoma	IV	G34R	p.R1739X %	T256fs	WT	WT	WT	WT	WT	WT	WT	WT	WT	WT
**PGBM 14**	Yes	Pediatric	15	Temporo-parietal lobe	M	Glioblastoma	IV	G34R	p.E1757X %	R273C; R248Q	WT	WT	WT	WT	WT	WT	WT	WT	WT	WT
**PGBM 15**	Yes	Pediatric	13	NA	M	Glioblastoma	IV	G34R	p.H2254R %	S51delins	WT	WT	WT	WT	WT	WT	WT	WT	WT	WT
**PGBM 16**	Yes	Pediatric	20	Parieto-occipital lobe	F	Glioblastoma	IV	G34V	p.R2111X %	R342X	Y183C $	WT	KDM2B: p.R610L	WT	WT	WT	WT	WT	WT	WT
**PGBM 17**	Yes	Pediatric	17	Frontal lobe	M	Glioblastoma	IV	WT	p.G1589V %	Y220C	R132H	WT	WT	WT	WT	WT	WT	WT	WT	WT
**PGBM 18**	Yes	Pediatric	14	Temporal lobe	M	Glioblastoma	IV	WT	p.R1426X %	R273C; R196X	R132H	p.R2109X	WT	WT	WT	WT	p.H83Y	MSH6: p.R482X, MSH6: p.F342L	WT	p.R88Q; p.G106V
**PGBM 19**	Yes	Pediatric	20	NA	M	Glioblastoma	IV	WT	p.K1584fs (ATRX); p.C629fs (DAXX)	R267Q; T230I	WT	WT	WT	WT	WT	WT	WT	WT	WT	WT
**PGBM 20**	Yes	Pediatric	11	NA	M	Glioblastoma	IV	WT	p.N2443D	R248W	WT	WT	WT	WT	WT	WT	WT	WT	WT	WT
**PGBM 21**	Yes	Pediatric	14	Temporal lobe	F	Glioblastoma	IV	WT	p.R238X (DAXX)	R267W; P152L	WT	WT	WT	WT	WT	WT	p.R22P	MSH6: p.R1035X	WT	WT
**PGBM 22**	Yes	Pediatric	17	Temporal lobe	M	Glioblastoma	IV	WT	p.R1302fs; p.D2098N; p.K1584fs	R337C; R175H	WT	p.(T305Qfs*35)	ASH1L p.G1520R; ASH1L p.R1321X	p.A722V	WT	WT	WT	MSH2: p.R406X	WT	WT
**PGBM 23**	Yes	Pediatric	13	NA	M	Glioblastoma	IV	WT	WT	I254S	R132H	WT	WT	WT	WT	WT	WT	WT	WT	WT
**PGBM 24**	Yes	Pediatric	14	NA	M	Glioblastoma	IV	WT	WT	R196X	WT	WT	WT	WT	WT	WT	CDKN2A deletion	WT	WT	WT
**PGBM 25**	Yes	Pediatric	12	Temporal lobe	M	Glioblastoma	IV	WT	WT	R342X	WT	p.P1470S	SETD3: p.A269T, KDM4B: p.R835C	WT	WT	WT	CDKN2A deletion	WT	WT	WT
**PGBM 26**	Yes	Pediatric	14	Frontal lobe	M	Glioblastoma	IV	WT	WT	R175H	WT	WT	WT	WT	WT	WT	WT	WT	WT	p.E545K
**PGBM 27**	Yes	Pediatric	9	Temporal lobe	F	Glioblastoma	IV	WT	WT	I251L	WT	WT	KDM5C: p.R323Q	EGFR Amp; p.D984N	WT	WT	WT	MSH2: p.N139S	WT	WT
**PGBM 29**	Yes	Pediatric	15	NA	M	Glioblastoma	IV	WT	p.H827Q	V10G	R132H	WT	WT	WT	WT	WT	WT	WT	WT	p.H1047R
**PGBM 30**	Yes	Pediatric	6	Thalamus	M	Glioblastoma	IV	WT	WT	G245S	WT	WT	KDM4A: p.R498C	WT	WT	WT	WT	WT	WT	WT
**PGBM 31**	Yes	Pediatric	7	NA	F	Glioblastoma	IV	WT	WT	WT	WT	WT	WT	WT	WT	WT	WT	WT	WT	WT
**PGBM 32**	Yes	Pediatric	4	Parietal lobe	M	Glioblastoma	IV	WT	WT	WT	WT	WT	WT	WT	WT	WT	WT	WT	WT	WT
**PGBM 33**	Yes	Pediatric	12	NA	M	Glioblastoma	IV	WT	WT	WT	WT	WT	WT	WT	WT	WT	WT	WT	WT	WT
**PGBM 34**	Yes	Pediatric	12	Parietal lobe	F	Glioblastoma	IV	WT	WT	WT	WT	p.(S1002Ffs*4)	KDM5C: p.D1040G	WT	WT	WT	CDKN2A deletion	WT	WT	WT
**PGBM 35**	Yes	Pediatric	7	Parietal lobe	M	Glioblastoma	IV	WT	WT	WT	WT	WT	WT	WT	p.V600E	WT	WT	WT	WT	WT
**PGBM 37**	Yes	Pediatric	7	Cerebellum	M	Glioblastoma	IV	WT	WT	WT	WT	WT	WT	WT	WT	WT	WT	WT	WT	WT
**PGBM 39**	Yes	Pediatric	12	Parietal lobe	F	Glioblastoma	IV	WT	WT	WT	WT	WT	WT	WT	p.V600E	WT	CDKN2A deletion	WT	WT	WT
**PGBM 40**	Yes	Pediatric	14	Thalamus	F	Glioblastoma	IV	WT	WT	WT	WT	WT	WT	WT	WT	WT	WT	MSH2: p.S860L &	WT	WT
**PGBM 41**	Yes	Pediatric	7	Thalamus	F	Glioblastoma	IV	WT	WT	WT	WT	WT	WT	WT	WT	WT	WT	WT	WT	WT
**PGBM 42**	Yes	Pediatric	2	NA	F	Glioblastoma	IV	WT	WT	WT	WT	WT	KDM2B: p.G250D, KDM4A: p.R518W	WT	WT	WT	WT	WT	WT	WT
**PGBM 43**	Yes	Pediatric	16	Frontal lobe	F	Glioblastoma-recurrence	IV	WT	WT	WT	WT	WT	WT	WT	WT	WT	WT	WT	WT	WT
**PGBM 44**	Yes	Pediatric	16	Cortex	F	Glioblastoma	IV	WT	WT	WT	WT	WT	WT	WT	WT	WT	WT	WT	WT	WT
**PGBM 46**	Yes	Pediatric	14	NA	M	Glioblastoma	IV	WT	WT	WT	WT	WT	WT	WT	WT	WT	WT	WT	WT	WT
**PGBM 47**	Yes	Pediatric	14	NA	F	Glioblastoma	IV	WT	WT	WT	WT	WT	WT	WT	p.V600E	WT	WT	WT	WT	WT
**PGBM 48**	Yes	Pediatric	2	NA	M	Glioblastoma	IV	WT	WT	WT	WT	WT	WT	WT	WT	WT	WT	WT	WT	WT
**PGBM 49**	Yes	Pediatric	5.4	Frontal lobe	M	Glioblastoma	IV	WT	WT	WT	WT	WT	WT	WT	WT	WT	WT	WT	WT	WT
**PGBM 50**	Yes	Pediatric	1	Cortex	F	Glioblastoma	IV	WT	WT	WT	WT	WT	WT	WT	WT	WT	WT	WT	WT	WT
**PGBM 51**	Yes	Pediatric	15	Frontal lobe	M	Glioblastoma	IV	WT	p.H2214R	R141C	R132H	NM_014159.6:c.6293+1G>A	PBRM1: p.V49M, UTX/KDM6A: p.N366S	WT	WT	WT	WT	MSH6: p.T1085fs	WT	p.R93Q
**PGBM 52**	Yes	Pediatric	5	NA	F	Glioblastoma	IV	WT	WT	WT	WT	WT	WT	WT	WT	WT	WT	WT	WT	WT
**PGBM 53**	Yes	Pediatric	NA	NA	F	Glioblastoma	IV	K27M	WT	p.C3Y	WT	WT	WT	WT	WT	WT	WT	WT	WT	WT
**PGBM 54**	Yes	Pediatric	17	Frontal lobe	F	Glioblastoma	IV	WT	WT	p.H47R	R132H	WT	WT	WT	WT	WT	WT	WT	WT	WT
**PGBM 55**	No	Pediatric	18	Temporal lobe	M	Glioblastoma	IV	NA	NA	NA	NA	p.E498X	NA	NA	NA	NA	NA	NA	NA	NA
**PGBM 56**	No	Pediatric	6	Thalamus	F	Glioblastoma	IV	K27M	NA	NA	WT	WT	NA	NA	NA	NA	NA	NA	NA	NA
**PGBM 57**	No	Pediatric	11	Thalamus	M	Glioblastoma	IV	K27M	NA	NA	WT	WT	NA	NA	NA	NA	NA	NA	NA	NA
**PGBM 58**	No	Pediatric	14	Temporal lobe	M	Glioblastoma	IV	WT	NA	NA	R132H	WT	NA	NA	NA	NA	NA	NA	NA	NA
**PGBM 59**	No	Pediatric	12	Frontal lobe	M	Glioblastoma	IV	WT	NA	NA	R132H	p.1385_1385del, p.R2490Q	NA	NA	WT	NA	NA	NA	NA	NA
**PGBM 60**	Yes	Pediatric	9	Temporal lobe	M	Glioblastoma	IV	WT	p.S2146L	WT	WT	WT	WT	WT	WT	WT	WT	WT	WT	WT
**PGBM 61**	No	Pediatric	17	Parieto-occipital lobe	F	Glioblastoma	IV	G34R	NA	NA	WT	WT	NA	NA	WT	NA	NA	NA	NA	NA
**PGBM 62**	No	Pediatric	16	Spinal cord	M	Glioblastoma	IV	K27M	NA	NA	NA	p.R329Q (not conserved) #	NA	NA	NA	NA	NA	NA	NA	NA
**PGBM 63**	No	Pediatric	16	NA	F	Glioblastoma	IV	NA	NA	NA	NA	WT	NA	NA	NA	NA	NA	NA	NA	NA
**PGBM 64**	No	Pediatric	1	Frontal lobe	M	Glioblastoma	IV	NA	NA	NA	NA	p.R1686Q	NA	NA	NA	NA	NA	NA	NA	NA
**GLIOMA 001**	No	Adult	60	NA	F	Glioblastoma	IV	WT	NA	NA	WT	WT	NA	NA	NA	NA	NA	NA	NA	NA
**GLIOMA 002**	No	Adult	73	NA	F	Glioblastoma	IV	WT	NA	NA	WT	WT	NA	NA	NA	NA	NA	NA	NA	NA
**GLIOMA 003**	No	Adult	50	Parietal lobe	F	Glioblastoma	IV	WT	NA	NA	WT	p.S1660X	NA	NA	NA	NA	NA	NA	NA	NA
**GLIOMA 004**	No	Adult	45	NA	F	Glioblastoma	IV	WT	NA	NA	R132H	WT	NA	NA	NA	NA	NA	NA	NA	NA
**GLIOMA 005**	No	Adult	80	NA	F	Glioblastoma	IV	WT	NA	NA	WT	WT	NA	NA	NA	NA	NA	NA	NA	NA
**GLIOMA 006**	No	Adult	34	NA	M	Glioblastoma	IV	WT	NA	NA	R132H	WT	NA	NA	NA	NA	NA	NA	NA	NA
**GLIOMA 007**	No	Adult	72	NA	F	Glioblastoma	IV	WT	NA	NA	WT	WT	NA	NA	NA	NA	NA	NA	NA	NA
**GLIOMA 008**	No	Adult	73	NA	F	Glioblastoma	IV	WT	NA	NA	WT	WT	NA	NA	NA	NA	NA	NA	NA	NA
**GLIOMA 009**	No	Adult	62	NA	F	Glioblastoma	IV	WT	NA	NA	WT	WT	NA	NA	NA	NA	NA	NA	NA	NA
**GLIOMA 010**	No	Adult	53	Parietal lobe	M	Glioblastoma-recurrence	IV	WT	WT	NA	WT	p.D868G	NA	NA	NA	NA	NA	NA	NA	NA
**GLIOMA 011**	No	Adult	75	NA	M	Glioblastoma	IV	WT	NA	NA	WT	WT	NA	NA	NA	NA	NA	NA	NA	NA
**GLIOMA 012**	No	Adult	70	NA	F	Glioblastoma	IV	WT	NA	NA	WT	WT	NA	NA	NA	NA	NA	NA	NA	NA
**GLIOMA 013**	No	Adult	65	NA	F	Glioblastoma	IV	WT	NA	NA	WT	WT	NA	NA	NA	NA	NA	NA	NA	NA
**GLIOMA 014**	No	Adult	66	NA	F	Glioblastoma	IV	WT	NA	NA	WT	WT	NA	NA	NA	NA	NA	NA	NA	NA
**GLIOMA 015**	No	Adult	53	NA	F	Glioblastoma	IV	WT	NA	NA	WT	WT	NA	NA	NA	NA	NA	NA	NA	NA
**GLIOMA 016**	No	Adult	49	NA	F	Glioblastoma	IV	WT	NA	NA	WT	WT	NA	NA	NA	NA	NA	NA	NA	NA
**GLIOMA 017**	No	Adult	72	NA	M	Glioblastoma	IV	WT	NA	NA	WT	WT	NA	NA	NA	NA	NA	NA	NA	NA
**GLIOMA 018**	No	Adult	74	NA	M	Glioblastoma-recurrence	IV	WT	NA	NA	WT	WT	NA	NA	NA	NA	NA	NA	NA	NA
**GLIOMA 019**	No	Adult	72	NA	F	Glioblastoma	IV	WT	NA	NA	WT	WT	NA	NA	NA	NA	NA	NA	NA	NA
**GLIOMA 020**	No	Adult	60	NA	M	Glioblastoma	IV	WT	NA	NA	WT	WT	NA	NA	NA	NA	NA	NA	NA	NA
**GLIOMA 021**	No	Adult	67	NA	F	Glioblastoma	IV	WT	NA	NA	WT	WT	NA	NA	NA	NA	NA	NA	NA	NA
**GLIOMA 022**	No	Adult	64	NA	M	Glioblastoma-recurrence	IV	WT	NA	NA	WT	WT	NA	NA	NA	NA	NA	NA	NA	NA
**GLIOMA 023**	No	Adult	64	NA	M	Glioblastoma	IV	WT	NA	NA	WT	WT	NA	NA	NA	NA	NA	NA	NA	NA
**GLIOMA 024**	No	Adult	46	NA	F	Glioblastoma	IV	WT	NA	NA	R132H	WT	NA	NA	NA	NA	NA	NA	NA	NA
**GLIOMA 025**	No	Adult	48	Occipital lobe	M	Glioblastoma	IV	WT	WT	WT	WT	p.(K853Rfs*37)	NA	NA	NA	NA	NA	NA	NA	NA
**GLIOMA 026**	No	Adult	22	NA	M	Glioblastoma	IV	K27M	NA	NA	WT	WT	NA	NA	NA	NA	NA	NA	NA	NA
**GLIOMA 027**	No	Adult	38	NA	M	Glioblastoma	IV	WT	NA	NA	WT	WT	NA	NA	NA	NA	NA	NA	NA	NA
**GLIOMA 028**	No	Adult	42	NA	M	Glioblastoma	IV	WT	NA	NA	WT	WT	NA	NA	NA	NA	NA	NA	NA	NA
**GLIOMA 029**	No	Adult	42	NA	M	Glioblastoma	IV	WT	NA	NA	R132H	WT	NA	NA	NA	NA	NA	NA	NA	NA
**GLIOMA 030**	No	Adult	43	NA	M	Glioblastoma	IV	WT	NA	NA	WT	WT	NA	NA	NA	NA	NA	NA	NA	NA
**GLIOMA 031**	No	Adult	44	NA	M	Glioblastoma	IV	WT	NA	NA	WT	WT	NA	NA	NA	NA	NA	NA	NA	NA
**GLIOMA 032**	No	Adult	46	NA	F	Glioblastoma	IV	WT	NA	NA	WT	WT	NA	NA	NA	NA	NA	NA	NA	NA
**GLIOMA 033**	No	Adult	46	NA	M	Glioblastoma	IV	WT	NA	NA	WT	WT	NA	NA	NA	NA	NA	NA	NA	NA
**GLIOMA 034**	No	Adult	47	NA	F	Glioblastoma	IV	WT	NA	NA	R132H	WT	NA	NA	NA	NA	NA	NA	NA	NA
**GLIOMA 035**	No	Adult	48	NA	F	Glioblastoma	IV	WT	NA	NA	R132H	WT	NA	NA	NA	NA	NA	NA	NA	NA
**GLIOMA 036**	No	Adult	51	NA	M	Glioblastoma	IV	WT	NA	NA	R132H	WT	NA	NA	NA	NA	NA	NA	NA	NA
**GLIOMA 037**	No	Adult	42	NA	F	Glioblastoma	IV	WT	NA	NA	WT	WT	NA	NA	NA	NA	NA	NA	NA	NA
**GLIOMA 038**	No	Adult	66	NA	M	Anaplastic astrocytoma	III	WT	NA	NA	WT	WT	NA	NA	NA	NA	NA	NA	NA	NA
**GLIOMA 039**	No	Pediatric	17	Cerebello-pontine angle	M	Anaplastic astrocytoma	III	K27M	NA	NA	WT	WT	NA	NA	NA	NA	NA	NA	NA	NA
**GLIOMA 040**	No	Pediatric	17	Frontal lobe	M	Anaplastic astrocytoma	III	WT	NA	NA	R132C	WT	NA	NA	WT	NA	NA	NA	NA	NA
**GLIOMA 041**	No	Pediatric	15	Temporal lobe	F	Oligoastrocytoma III	III	WT	NA	NA	WT	WT	NA	NA	NA	NA	NA	NA	NA	NA
**GLIOMA 042**	Yes	Pediatric	3	NA	F	Anaplastic astrocytoma	III	WT	WT	NA	WT	WT	WT	WT	WT	WT	WT	WT	WT	WT
**GLIOMA 043**	No	Adult	42	NA	F	Oligoastrocytoma III	III	WT	NA	NA	R132H	WT	NA	NA	NA	NA	NA	NA	NA	NA
**GLIOMA 044**	No	Pediatric	16	Frontal lobe	M	Anaplastic astrocytoma	III	WT	NA	NA	WT	WT	NA	NA	WT	NA	NA	NA	NA	NA
**GLIOMA 045**	No	Adult	64	NA	F	Oligoastrocytoma III	III	WT	NA	NA	R132H	WT	NA	NA	NA	NA	NA	NA	NA	NA
**GLIOMA 046**	No	Adult	58	NA	M	Oligoastrocytoma III	III	WT	NA	NA	R132H	WT	NA	NA	NA	NA	NA	NA	NA	NA
**GLIOMA 047**	No	Adult	37	NA	M	Oligoastrocytoma III	III	WT	NA	NA	R132H	WT	NA	NA	NA	NA	NA	NA	NA	NA
**GLIOMA 048**	No	Adult	37	NA	M	Oligoastrocytoma III	III	WT	NA	NA	R132H	WT	NA	NA	NA	NA	NA	NA	NA	NA
**GLIOMA 049**	No	Adult	34	NA	M	Oligoastrocytoma III-recurrence	III	WT	NA	NA	R132H	WT	NA	NA	NA	NA	NA	NA	NA	NA
**GLIOMA 050**	No	Adult	37	NA	M	Oligoastrocytoma III	III	WT	NA	NA	R132H	WT	NA	NA	NA	NA	NA	NA	NA	NA

**GLIOMA 051**	No	Adult	48	NA	M	Oligodendroglioma III	III	WT	NA	NA	R132H	WT	NA	NA	NA	NA	NA	NA	NA	NA
**GLIOMA 052**	No	Adult	69	NA	M	Oligodendroglioma III	III	WT	NA	NA	WT	WT	NA	NA	NA	NA	NA	NA	NA	NA
**GLIOMA 053**	No	Adult	48	NA	M	Oligodendroglioma III	III	WT	NA	NA	R132H	WT	NA	NA	NA	NA	NA	NA	NA	NA
**GLIOMA 054**	No	Adult	32	NA	M	Oligodendroglioma III-recurrence	III	WT	NA	NA	R132H	WT	NA	NA	NA	NA	NA	NA	NA	NA
**GLIOMA 055**	No	Adult	44	NA	F	Oligoastrocytoma III	III	WT	NA	NA	R132H	WT	NA	NA	NA	NA	NA	NA	NA	NA
**GLIOMA 056**	No	Adult	54	NA	F	Oligodendroglioma III	III	WT	NA	NA	R132H	WT	NA	NA	NA	NA	NA	NA	NA	NA
**GLIOMA 057**	No	Adult	35	NA	M	Oligodendroglioma III	III	WT	NA	NA	R132H	WT	NA	NA	NA	NA	NA	NA	NA	NA
**GLIOMA 058**	No	Adult	58	NA	M	Oligodendroglioma III-recurrence	III	WT	NA	NA	R132H	WT	NA	NA	NA	NA	NA	NA	NA	NA
**GLIOMA 059**	No	Adult	51	NA	M	Oligodendroglioma III	III	WT	NA	NA	R132H	WT	NA	NA	NA	NA	NA	NA	NA	NA
**GLIOMA 060**	No	Adult	52	NA	F	Oligoastrocytoma III	III	WT	NA	NA	NA	WT	NA	NA	NA	NA	NA	NA	NA	NA
**GLIOMA 061**	No	Adult	41	NA	F	Oligodendroglioma III	III	WT	NA	NA	R132H	WT	NA	NA	NA	NA	NA	NA	NA	NA
**GLIOMA 062**	No	Adult	58	Insular cortex	F	Oligodendroglioma III	III	WT	NA	NA	WT	p.R1625C	NA	NA	NA	NA	NA	NA	NA	NA
**GLIOMA 063**	No	Adult	43	Temporal lobe	M	Oligodendroglioma III-recurrence	III	WT	NA	NA	R132H	p.(S342Qfs*17)	NA	NA	NA	NA	NA	NA	NA	NA
**GLIOMA 064**	No	Adult	41	NA	M	Oligodendroglioma III	III	WT	NA	NA	NA	WT	NA	NA	NA	NA	NA	NA	NA	NA
**GLIOMA 065**	No	Adult	52	NA	F	Oligodendroglioma III-recurrence	III	WT	NA	NA	NA	WT	NA	NA	NA	NA	NA	NA	NA	NA
**GLIOMA 066**	No	Adult	39	NA	M	Oligodendroglioma III	III	WT	NA	NA	NA	WT	NA	NA	NA	NA	NA	NA	NA	NA
**GLIOMA 067**	Yes	Pediatric	4	Frontal lobe	M	Oligodendroglioma III	III	WT	WT	WT	WT	WT	WT	WT	WT	WT	WT	WT	WT	WT
**GLIOMA 068**	Yes	Pediatric	5	NA	M	Anaplastic astrocytoma	III	WT	WT	WT	WT	WT	WT	WT	WT	WT	WT	WT	WT	WT
**GLIOMA 069**	Yes	Pediatric	5	Cerebellum	M	Anaplastic astrocytoma	III	WT	WT	WT	WT	WT	PBRM1: p.E1533D	WT	WT	WT	WT	WT	WT	WT
**GLIOMA 070**	Yes	Pediatric	13	Pineal region	F	Anaplastic astrocytoma	III	WT	WT	WT	WT	WT	WT	WT	WT	WT	WT	WT	WT	WT
**GLIOMA 071**	Yes	Pediatric	14	Temporal lobe	M	Anaplastic astrocytoma	III	WT	WT	WT	Y183C $	WT	WT	WT	p.V600E	WT	WT	WT	WT	WT
**GLIOMA 072**	Yes	Pediatric	17	Temporo-parietal lobe	F	Anaplastic astrocytoma	III	WT	WT	WT	WT	p.(K583Sfs*17)	WT	WT	WT	WT	WT	WT	WT	WT
**GLIOMA 073**	No	Adult	36	NA	NA	Anaplastic astrocytoma	III	WT	NA	NA	R132H	WT	NA	NA	NA	NA	NA	NA	NA	NA
**GLIOMA 074**	No	Adult	37	NA	M	Anaplastic astrocytoma	III	WT	NA	NA	WT	WT	NA	NA	NA	NA	NA	NA	NA	NA
**GLIOMA 075**	No	Adult	46	NA	F	Oligoastrocytoma III	III	WT	NA	NA	WT	WT	NA	NA	NA	NA	NA	NA	NA	NA
**GLIOMA 076**	No	Adult	60	NA	F	Anaplastic astrocytoma	III	WT	NA	NA	WT	WT	NA	NA	NA	NA	NA	NA	NA	NA
**GLIOMA 077**	Yes	Pediatric	9.6	Posterior fossa	F	Anaplastic astrocytoma	III	WT	WT	WT	WT	WT	SETD3: p.N331S	WT	p.A598delinsAT	WT	WT	WT	WT	WT
**GLIOMA 078**	No	Pediatric	5	Parietal lobe	M	Astrocytoma II	II	K27M	NA	NA	NA	WT	NA	NA	NA	NA	NA	NA	NA	NA
**GLIOMA 079**	No	Pediatric	15	Cerebellum	F	Astrocytoma II	II	WT	NA	NA	NA	WT	NA	NA	NA	NA	NA	NA	NA	NA
**GLIOMA 080**	No	Pediatric	3	Thalamus	F	Astrocytoma II	II	WT	NA	NA	NA	WT	NA	NA	NA	NA	NA	NA	NA	NA
**GLIOMA 081**	No	Pediatric	13	Bilocation frontal lobe and contralateral occipital lobe	M	Astrocytoma II	II	WT	NA	NA	NA	WT	NA	NA	NA	NA	NA	NA	NA	NA
**GLIOMA 082**	No	Adult	54	NA	M	Astrocytoma II	II	WT	NA	NA	NA	WT	NA	NA	NA	NA	NA	NA	NA	NA
**GLIOMA 083**	No	Adult	45	NA	F	Oligodendroglioma II	II	WT	NA	NA	NA	WT	NA	NA	NA	NA	NA	NA	NA	NA
**GLIOMA 084**	No	Adult	52	NA	M	Oligoastrocytoma II	II	WT	NA	NA	NA	WT	NA	NA	NA	NA	NA	NA	NA	NA
**GLIOMA 085**	No	Pediatric	3	Cerebellum	M	Oligoastrocytoma II	II	WT	NA	NA	NA	WT	NA	NA	NA	NA	NA	NA	NA	NA
**GLIOMA 086**	No	Adult	60	NA	F	Astrocytoma II	II	WT	NA	NA	NA	WT	NA	NA	NA	NA	NA	NA	NA	NA
**GLIOMA 087**	No	Pediatric	15	Cerebellum	M	Astrocytoma II	II	WT	NA	NA	NA	WT	NA	NA	NA	NA	NA	NA	NA	NA
**GLIOMA 088**	No	Pediatric	9	Frontal lobe	F	Astrocytoma II	II	WT	NA	NA	NA	WT	NA	NA	NA	NA	NA	NA	NA	NA
**GLIOMA 089**	No	Pediatric	8	Temporal lobe	M	Astrocytoma II	II	WT	NA	NA	NA	WT	NA	NA	NA	NA	NA	NA	NA	NA
**GLIOMA 090**	No	Pediatric	15	Parietal lobe	F	Astrocytoma II	II	WT	NA	NA	NA	WT	NA	NA	NA	NA	NA	NA	NA	NA
**GLIOMA 091**	No	Pediatric	4	Optic chiasma	F	Astrocytoma II	II	WT	NA	NA	WT	WT	NA	NA	NA	NA	NA	NA	NA	NA
**GLIOMA 092**	No	Pediatric	6.8	Posterior fossa	F	Astrocytoma II	II	WT	NA	NA	WT	WT	NA	NA	NA	NA	NA	NA	NA	NA
**GLIOMA 093**	Yes	Pediatric	12	Cortex	F	Oligodendroglioma/Oligoastrocytoma III	III	WT	WT	WT	Y183C $	p.G1621R	WT	p.P596L	WT	WT	WT	WT	WT	WT
**GLIOMA 094**	No	Adult	46	NA	F	Astrocytoma II	II	WT	NA	NA	R132H	WT	NA	NA	NA	NA	NA	NA	NA	NA
**GLIOMA 095**	No	Pediatric	2	Cerebellum	M	Astrocytoma II	II	WT	NA	NA	WT	WT	NA	NA	NA	NA	NA	NA	NA	NA
**GLIOMA 096**	No	Pediatric	3	Cerebellum	M	Astrocytoma II	II	WT	NA	NA	WT	WT	NA	NA	NA	NA	NA	NA	NA	NA
**GLIOMA 097**	No	Pediatric	9	Temporal lobe	M	Astrocytoma II	II	WT	NA	NA	WT	WT	NA	NA	NA	NA	NA	NA	NA	NA
**GLIOMA 098**	No	Pediatric	18	Frontal lobe	F	Astrocytoma II	II	WT	NA	NA	R132C	WT	NA	NA	NA	NA	NA	NA	NA	NA
**GLIOMA 099**	No	Pediatric	19	NA	F	Astrocytoma II	II	WT	NA	NA	R132S	WT	NA	NA	NA	NA	NA	NA	NA	NA
**GLIOMA 100**	No	Adult	44	NA	M	Astrocytoma II	II	WT	NA	NA	R132H	WT	NA	NA	NA	NA	NA	NA	NA	NA
**GLIOMA 101**	No	Adult	55	NA	M	Oligoastrocytoma II	II	WT	NA	NA	R132H	WT	NA	NA	NA	NA	NA	NA	NA	NA
**GLIOMA 102**	No	Pediatric	NA	Temporal lobe	NA	Astrocytoma II	II	WT	NA	NA	R132H	WT	NA	NA	NA	NA	NA	NA	NA	NA
**GLIOMA 103**	No	Pediatric	14	Cortex	F	Glioblastoma	IV	WT	NA	NA	WT	p.V2272A	NA	NA	WT	NA	NA	NA	NA	NA
**GLIOMA 104**	No	Adult	22	Left Frontal Cortex	F	Astrocytoma II	II	WT	NA	NA	R132H	WT	NA	NA	NA	NA	NA	NA	NA	NA
**GLIOMA 105**	No	Adult	21	Diencephalon, III ventricle	M	Astrocytoma II	II	WT	NA	NA	WT	WT	NA	NA	NA	NA	NA	NA	NA	NA
**GLIOMA 106**	No	Adult	70	Left Temporal Cortex	M	Astrocytoma II	II	WT	NA	NA	WT	WT	NA	NA	NA	NA	NA	NA	NA	NA
**GLIOMA 107**	No	Adult	37	Right Temporo-parietal Cortex	M	Astrocytoma II	II	WT	NA	NA	R132H	WT	NA	NA	NA	NA	NA	NA	NA	NA
**GLIOMA 108**	No	Adult	74	Right Parietal Cortex	F	Astrocytoma II	II	WT	NA	NA	WT	WT	NA	NA	NA	NA	NA	NA	NA	NA
**GLIOMA 109**	No	Adult	50	Left Temporal Cortex	M	Astrocytoma II	II	WT	NA	NA	WT	WT	NA	NA	NA	NA	NA	NA	NA	NA
**GLIOMA 110**	No	Adult	54	Right Temporo-parietal Cortex	M	Astrocytoma II	II	WT	NA	NA	WT	WT	NA	NA	NA	NA	NA	NA	NA	NA
**GLIOMA 111**	No	Adult	39	Right Parietal Cortex	F	Astrocytoma II	II	WT	NA	NA	R132H	WT	NA	NA	NA	NA	NA	NA	NA	NA
**GLIOMA 112**	No	Adult	44	Tectum (lamina quadrigemina)	F	Astrocytoma II	II	WT	NA	NA	WT	WT	NA	NA	NA	NA	NA	NA	NA	NA
**GLIOMA 113**	No	Adult	48	Left hypothalamus	F	Astrocytoma II	II	WT	NA	NA	WT	WT	NA	NA	NA	NA	NA	NA	NA	NA
**GLIOMA 114**	No	Adult	66	Left Frontal Cortex	F	Astrocytoma II	II	WT	NA	NA	WT	WT	NA	NA	NA	NA	NA	NA	NA	NA
**GLIOMA 115**	No	Adult	34	Left Temporal Cortex	M	Astrocytoma II	II	WT	NA	NA	R132H	WT	NA	NA	NA	NA	NA	NA	NA	NA
**GLIOMA 116**	No	Adult	29	Right Frontal Cortex	F	Astrocytoma II	II	WT	NA	NA	R132H	WT	NA	NA	NA	NA	NA	NA	NA	NA
**GLIOMA 117**	No	Adult	53	Right Temporal Cortex	M	Astrocytoma II	II	WT	NA	NA	R132H	WT	NA	NA	NA	NA	NA	NA	NA	NA
**GLIOMA 118**	No	Pediatric	2	Thalamus	M	Astrocytoma II	II	WT	NA	NA	WT	WT	NA	NA	NA	NA	NA	NA	NA	NA
**GLIOMA 119**	No	Pediatric	4	Brainstem	M	Astrocytoma II	II	WT	NA	NA	WT	WT	NA	NA	NA	NA	NA	NA	NA	NA
**GLIOMA 120**	No	Pediatric	4	Cerebellar Hemisphere	F	Astrocytoma II	II	WT	NA	NA	WT	WT	NA	NA	NA	NA	NA	NA	NA	NA
**GLIOMA 121**	No	Pediatric	13	Brainstem	M	Astrocytoma II	II	WT	NA	NA	WT	WT	NA	NA	NA	NA	NA	NA	NA	NA
**GLIOMA 122**	No	Pediatric	15	Pineal Region	F	Astrocytoma II	II	WT	NA	NA	WT	WT	NA	NA	NA	NA	NA	NA	NA	NA
**GLIOMA 123**	No	Adult	46	NA	M	Oligodendroglioma II	II	WT	NA	NA	R132H	WT	NA	NA	NA	NA	NA	NA	NA	NA
**GLIOMA 124**	No	Pediatric	0.4	Cerebellar Vermis	M	Astrocytoma II	II	WT	NA	NA	WT	WT	NA	NA	NA	NA	NA	NA	NA	NA

% Homozygous mutations.
$ This variant is not predicted to be damaging as it arises in control samples.
# This amino acid substitution is not predicted to be damaging as it is not at a conserved residue.
*Samples with a private mutation in either *ASH1L, SETD3, PBRM1, KDM2A, KDM2B, KDM4A, KDM4B, KDM4C, KDM5C, UTX/KDM6A.*
§ Samples demonstrating a private variant that is not highly conserved or with an amino acid change that has been seen in other vertebrates is indicated, but not highlighted within the table. These variants are highlighted as red text when occurring concurrently with potentially damaging variants.
£ Samples demonstrating homozygous loss at chromosomal region 9p21 encompassing the *CDKN2A* locus indicated as "*CDKN2A* deletion", previously published in Schwartzentruber *et al* (2012) *Nature* Jan 29;482(7384):226-31. doi: 10.1038/nature10833.
¥ Samples demonstrating high-level focal amplification at the *EGFR* locus at 7p12 indicated as "EGFR amp," previously published in Schwartzentruber *et al* (2012) *Nature* Jan 29;482(7384):226-31. doi: 10.1038/nature10833.
& This variant is not well-conserved across vertebrates however is present within the germline of this sample.
