# Supplementary material for: Mutations in SETD2 and genes affecting histone H3K36 methylation target hemispheric high-grade gliomas
Source: Acta Neuropathol. 2013 Feb 16;125(5):659–69. doi: 10.1007/s00401-013-1095-8 (PMC3631313; doi:10.1007/s00401-013-1095-8)
Supplement: Supplementary file 2 — Supplementary material 2 (PDF 51 kb) [file 401_2013_1095_MOESM2_ESM.pdf]

Table S2: Genes significantly mutated in 60 pediatric high-grade glioma samples

The number of genes of interest is used in calculating the FDR Q values.
Q values calculated as: q = p * numGenes / rank
Number of genes with >= 1 mutation: 6365
Number of genes of interest: 6365

	All mutations						
Gene	Raw p value	Rank	Q value	#Mutations ir	#Affecteds	#Mutations ir	#Controls
TP53	3.7014E-26	1	2.356E-22	27	60	4	543
H3F3A	2.43011E-15	2	7.734E-12	15	60	1	543
ATRX	5.67151E-12	3	1.203E-08	15	60	6	543
NF1	4.70528E-08	4	7.487E-05	12	60	9	543
**SETD2**	**2.25749E-05**	**5**	**0.0287378**	**8**	**60**	**7**	**543**
CSMD3	2.96158E-05	6	0.0314175	10	60	14	543
MBL2	8.9406E-05	7	0.0812956	4	60	0	543
HSP90B1	8.9406E-05	7	0.0812956	4	60	0	543
PITRM1	0.00011692	9	0.0826884	6	60	4	543
ASXL1	0.000149665	10	0.0952616	5	60	2	543
TRIM60	0.000149665	10	0.0952616	5	60	2	543
ATP2B1	0.000368577	12	0.1954995	5	60	3	543
TTN	0.000368877	13	0.1806079	25	60	111	543
PIK3CA	0.000413596	14	0.1880384	4	60	1	543
PRKAG1	0.000413596	14	0.1880384	4	60	1	543
CHD7	0.000765931	16	0.3046969	5	60	4	543
C18orf34	0.000765931	16	0.3046969	5	60	4	543
COL11A2	0.000907562	18	0.3209238	7	60	11	543
TUBB2B	0.000941116	19	0.3152737	3	60	0	543
CDHR4	0.000941116	19	0.3152737	3	60	0	543
ZNF668	0.000941116	19	0.3152737	3	60	0	543
NINJ2	0.000941116	19	0.3152737	3	60	0	543
LILRB2	0.000941116	19	0.3152737	3	60	0	543
PDCL2	0.000941116	19	0.3152737	3	60	0	543
RPE65	0.000941116	19	0.3152737	3	60	0	543
ENOX1	0.000941116	19	0.3152737	3	60	0	543
SUCNR1	0.000941116	19	0.3152737	3	60	0	543
HS1BP3	0.001148174	28	0.2610045	4	60	2	543
CHPF2	0.001148174	28	0.2610045	4	60	2	543
ITGA1	0.001148174	28	0.2610045	4	60	2	543
SYNPO2	0.001148174	28	0.2610045	4	60	2	543
LIG1	0.001414942	32	0.2814408	5	60	5	543
ODZ4	0.001836634	33	0.3542477	9	60	22	543
KIAA1244	0.001863277	34	0.3488163	6	60	9	543
PRR21	0.001863277	34	0.3488163	6	60	9	543
LTBP1	0.002396302	36	0.4236795	5	60	6	543
LTF	0.002396302	36	0.4236795	5	60	6	543
ZNF697	0.002479519	38	0.4153194	4	60	3	543
PLCG2	0.002479519	38	0.4153194	4	60	3	543
FBXW7	0.002479519	38	0.4153194	4	60	3	543
CDH1	0.002479519	38	0.4153194	4	60	3	543
LARP7	0.003496245	42	0.5298475	3	60	1	543
RHOBTB1	0.003496245	42	0.5298475	3	60	1	543
DNASE2B	0.003496245	42	0.5298475	3	60	1	543
HAL	0.003496245	42	0.5298475	3	60	1	543
GNL1	0.003496245	42	0.5298475	3	60	1	543
LATS1	0.003496245	42	0.5298475	3	60	1	543
FGL1	0.003496245	42	0.5298475	3	60	1	543
JAK1	0.003496245	42	0.5298475	3	60	1	543
HAPLN1	0.003496245	42	0.5298475	3	60	1	543
SLC35A5	0.003496245	42	0.5298475	3	60	1	543
MARCH3	0.003496245	42	0.5298475	3	60	1	543
REPIN1	0.003496245	42	0.5298475	3	60	1	543
ACTL7B	0.003496245	42	0.5298475	3	60	1	543
C14orf169	0.003496245	42	0.5298475	3	60	1	543
GPR15	0.003496245	42	0.5298475	3	60	1	543

The number of genes of interest is used in calculating the FDR Q values.
Q values calculated as: q = p * numGenes / rank
#Genes with >= 1 TRUNC mutation: 835
Number of genes of interest: 835

	Truncating mutations						
Gene	Raw p value	Rank	Q value	#Mutations ir	#Affecteds	#Mutations ir	#Controls
NF1	2.60584E-10	1	2.17587E-07	11	60	2	543
TP53	4.71267E-10	2	1.96754E-07	10	60	1	543
ATRX	2.36697E-07	3	6.58808E-05	8	60	2	543
**SETD2**	**8.35849E-06**	**4**	**0.001744835**	**5**	**60**	**0**	**543**
AGL	0.000413596	5	0.069070525	4	60	1	543
C3orf32	0.008120217	6	0.602963765	3	60	2	543
OR2A7	0.009751905	7	0.602963765	2	60	0	543
HIP1R	0.009751905	7	0.602963765	2	60	0	543
FAM13B	0.009751905	7	0.602963765	2	60	0	543
DNASE2B	0.009751905	7	0.602963765	2	60	0	543
KIAA1217	0.009751905	7	0.602963765	2	60	0	543
BRAT1	0.009751905	7	0.602963765	2	60	0	543
PLEKHN1	0.009751905	7	0.602963765	2	60	0	543
FRK	0.009751905	7	0.602963765	2	60	0	543
CD1E	0.009751905	7	0.602963765	2	60	0	543
FGL1	0.009751905	7	0.602963765	2	60	0	543
MSH6	0.009751905	7	0.602963765	2	60	0	543
DOCK1	0.009751905	7	0.602963765	2	60	0	543
ALDH16A1	0.009751905	7	0.602963765	2	60	0	543
SYTL2	0.009751905	7	0.602963765	2	60	0	543
FBXW7	0.009751905	7	0.602963765	2	60	0	543
TM4SF1	0.009751905	7	0.602963765	2	60	0	543
REPIN1	0.009751905	7	0.602963765	2	60	0	543
SLCO1B3	0.009751905	7	0.602963765	2	60	0	543
TRIM60	0.009751905	7	0.602963765	2	60	0	543
ZNF343	0.009751905	7	0.602963765	2	60	0	543
BCOR	0.009751905	7	0.602963765	2	60	0	543
LAMA5	0.009751905	7	0.602963765	2	60	0	543
ZEB2	0.009751905	7	0.602963765	2	60	0	543
WDR41	0.009751905	7	0.602963765	2	60	0	543
KRTAP10-1	0.009751905	7	0.602963765	2	60	0	543
PRDM7	0.009751905	7	0.602963765	2	60	0	543
SSPO	0.023878862	33	0.602963765	4	60	8	543
TEX15	0.024551818	34	0.602963765	3	60	4	543
ANO5	0.027373484	35	0.653053107	2	60	1	543
NDRG1	0.027373484	35	0.653053107	2	60	1	543
PRR21	0.027373484	35	0.653053107	2	60	1	543
ARHGEF16	0.027373484	35	0.653053107	2	60	1	543
G6PC2	0.027373484	35	0.653053107	2	60	1	543
RB1	0.027373484	35	0.653053107	2	60	1	543
COL21A1	0.027373484	35	0.653053107	2	60	1	543
TSC2	0.027373484	35	0.653053107	2	60	1	543
ZNF761	0.027373484	35	0.653053107	2	60	1	543
C9orf174	0.027373484	35	0.653053107	2	60	1	543
MGAM	0.051250723	45	0.95098563	2	60	2	543
IQCE	0.051250723	45	0.95098563	2	60	2	543
ZNF560	0.051250723	45	0.95098563	2	60	2	543
FLT3	0.051250723	45	0.95098563	2	60	2	543
ZNF337	0.051250723	45	0.95098563	2	60	2	543
BRCA2	0.080004393	50	1	2	60	3	543
FBN3	0.080004393	50	1	2	60	3	543
MYBPC2	0.080004393	50	1	2	60	3	543
