# Supplementary material for: Mutations in SETD2 and genes affecting histone H3K36 methylation target hemispheric high-grade gliomas
Source: Acta Neuropathol. 2013 Feb 16;125(5):659–69. doi: 10.1007/s00401-013-1095-8 (PMC3631313; doi:10.1007/s00401-013-1095-8)
Supplement: Supplementary file 3 — Supplementary material 3 (PDF 116 kb) [file 401_2013_1095_MOESM3_ESM.pdf]

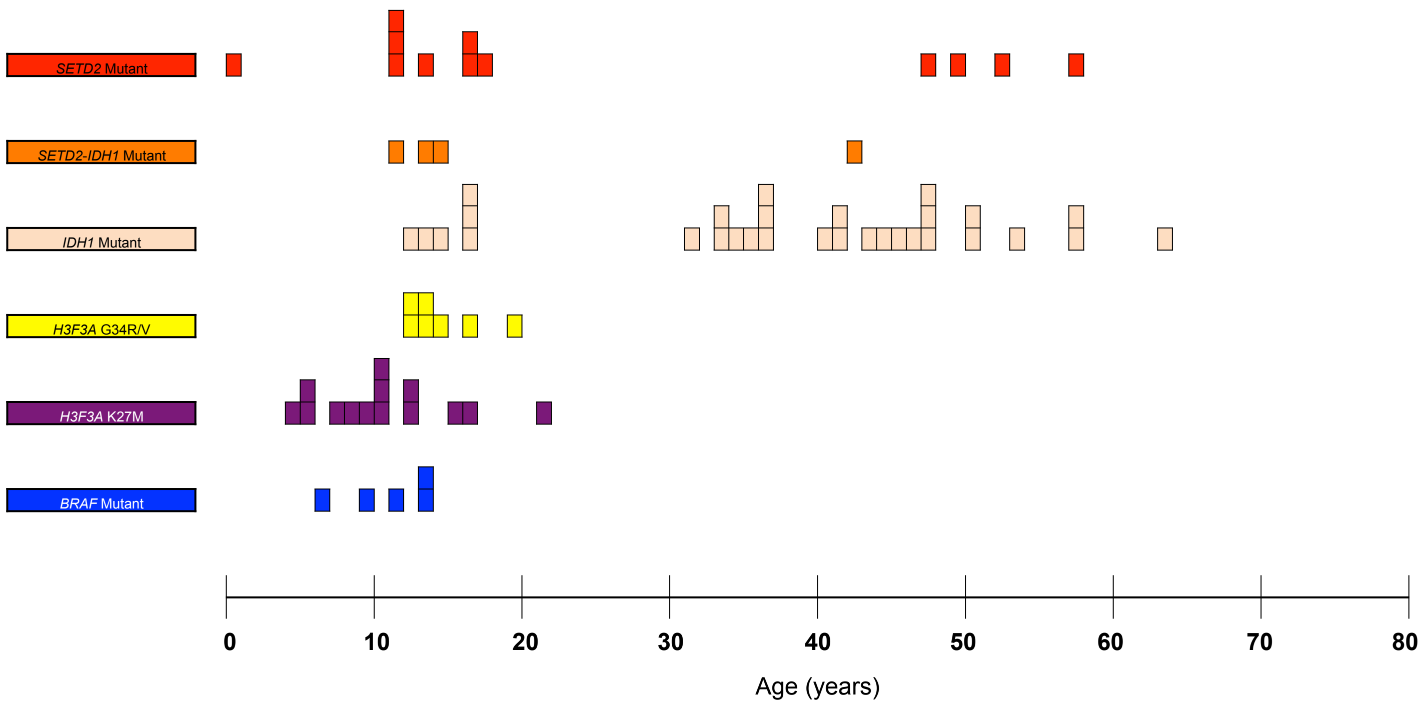

Supplementary Figure 1: Age distribution of driver mutations identified in pediatric and adult high-grade gliomas. Graphical representation of mutations identified in *SETD2*, *IDH1*, *H3F3A* (K27M, G34R/V) and *BRAF* in all pediatric and adult high-grade gliomas included in the study.

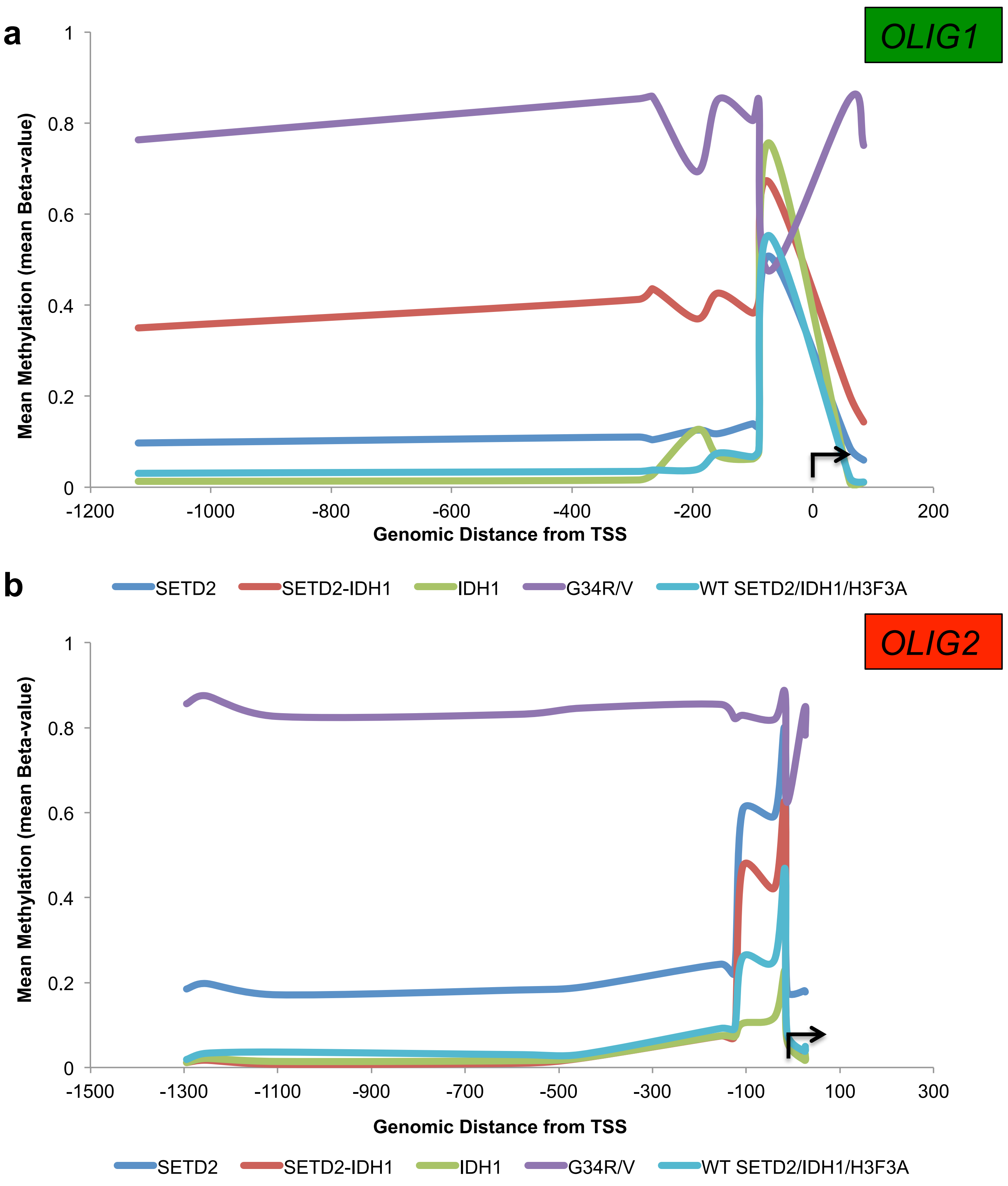

Supplementary Figure 2: ***SETD2 mutations do not show similar patterns of promoter methylation at OLIG1 and OLIG2 loci as H3.3K36 affected G34R/V tumors*.** **a,** Graphical representation of mean promoter methylation probe Beta-values within promoter region of *OLIG1* (**a**) and *OLIG2* (**b**). To best represent this region, probes were chosen that were within -1500bp upstream and +200bp downstream of the transcription start site (TSS).
